# Cost effectiveness of lanthanum carbonate in chronic kidney disease patients in Spain before and during dialysis

**DOI:** 10.1186/s13561-015-0049-3

**Published:** 2015-06-11

**Authors:** Blanca Gros, Antonio Galán, Emilio González-Parra, Jose A Herrero, Maria Echave, Stefan Vegter, Keith Tolley, Itziar Oyagüez

**Affiliations:** 1 Pharmacoeconomics & Outcomes Research Iberia (PORIB), Madrid, Spain; 2 Nephrology Department, Consorcio Hospital General Universitario, Valencia, Spain; 3 Nephrology Department, Hospital Fundación Jiménez Díaz, Madrid, Spain; 4 Nephrology Deparment, Hospital Clínico San Carlos, Madrid, Spain; 5 Department of Pharmacy, Unit of Pharmacoepidemiology and Pharmacoeconomics (PE), University ofGroningen, Groningen, The Netherlands; 6 Tolley Health Economics Ltd, Buxton, UK

**Keywords:** Hyperphosphatemia, Calcium binders, Lanthanum carbonate, Predialysis, Dialysis

## Abstract

**AIMS:**

In Spain, the first line treatment of hyperphosphatemia in Chronic Kidney Disease (CKD) consists of calcium-based phosphate binders (CB). However, their use is associated with vascular calcification and an increased mortality risk. The aim of this study was to assess the incremental cost-effectiveness of second-line Lanthanum Carbonate (LC) treatment in patients not responding to CB (calcium carbonate and calcium acetate).

**Material and methods:**

A lifetime Markov model was developed considering three health states (predialysis, dialysis and death). Transitions between states and efficacy data were obtained from randomized clinical trials and the European Dialysis and Transplant Association Annual report. Mortality rate was adjusted with the relative risk related to serum phosphorus levels.

According to the Spanish healthcare system perspective, only medical direct costs were considered. Dialysis costs (2013 prices in Euros) were obtained from diagnosis-related groups. Drug costs were derived from ex-factory prices, adjusted with 7.5% mandatory rebate. Quality of life estimates were based on a published systematic review. Costs and benefits were discounted at 3%. Deterministic and probabilistic sensitivity analyses (PSA) were conducted.

**Results:**

At the end of simulation, costs per patient with LC therapy were €1,169 and €5,044 with CB alone. 4.653 Quality Adjusted Life Years (QALYs) were gained per patient treated with LC, and 4.579 QALYs with CB. CB therapy is dominated by the LC strategy (i.e. lower costs, higher QALYs). Assuming a €30,000/QALY threshold, LC was dominant in 100% of PSA simulations.

**Conclusions:**

LC is a cost-effective second line treatment of hyperphosphatemia in CKD patients irrespective of dialysis status in Spain.

## Background

Chronic kidney disease (CKD) causes changes in calcium and phosphorus metabolism leading on hyperphosphatemia and hypercalcemia. During the last decade it has been demonstrated that elevated serum phosphorus (SP) and calcium levels may cause extraskeletal calcification of the tunica media in the vasculature of CKD patients [1]. These calcifications ultimately result in cardiovascular disease which is the leading cause of morbidity and mortality in patients with CKD [2].

Treatment guidelines from the Kidney Disease Outcomes Quality Initiative (K/DOQI) recommend that serum target levels be maintained between 3.5 and 5.5 mg/dL [3]. However. Spanish treatment guidelines recommend that SP be maintained under 4.5 mg/dL [4] and suggest to start the treatment for hyperphosphatemia, based in calcium-based phosphate binders (CB) (Calcium carbonate and calcium acetate), when dietary restrictions are insufficient [4]. However, it has been demonstrated that treatment with CB along with decreased of renal excretory capacity in CKD patients may accelerate the vascular calcification and increase cardiovascular mortality in the long term due to the calcium accumulation and/or the continuous hypercalcemia [5].

Second line treatment, after therapy failure with CB treatment, is based on the use of non-calcium binders including Lanthanum carbonate (LC). The efficacy of non-calcium binders has been demonstrated, resulting in the prevention and delay of CKD [6-9].

The cost-effectiveness of LC versus non-calcium binders for hyperphosphatemia in dialyzed patients has been demonstrated from UK, USA, Canadian and Japanese healthcare payer perspectives [10-13]. Cost-effectiveness of LC in second line treatment after CB has also been evaluated in predialysis from a UK perspective [14].

The aim of this study was to evaluate the cost-effectiveness of the use of LC as second line treatment in CKD patients in a Spanish healthcare context, irrespective of dialysis status, compared to a strategy of continued CB (calcium carbonate and calcium acetate) treatment.

## Methods

### Model description

A decision analytic model and Markov modeling techniques previously designed [14] were used to simulate the progression of a hypothetical cohort of 1,000 Spanish patients who are initially not on dialysis, representing the relevant stages of the natural history of the disease over time, as well as estimates for probability of progression between the health states related to CKD. All parameters of the model are summarized in Table 1.

**Table 1:** **Model inputs**

	**Predialysis value (95% CI)**	**Dialysis value (95% CI)**	
**Average patient age**	60 years		
**Gender**	60% male		
**Renal disease origin**	20% diabetes, 17% hypertension/renal vascular disease, 15% glomerulonephritis, 48% other cause		
**Target SP levels**	<4.6 mg/dL [3]		
**Initiation SP levels**	>4.6 mg/dL [19]	>5.5 mg/dL [20]	
**Drug efficacy**			
Response rate to CB	44.5% (32.1-57.1) [21]	34.1%(31.0-37.4) [22]	
Response rate to LC	38.3% (32.7-44.0) [19]	16.6% (13.5-19.9) [22]	
**Drug dosage**			
CC	3,000 mg/day	1,500 mg/day	
CA	5,000 mg/day	3,000 mg/day	
LC	1,875 mg/day	2,250 mg/day	
**Baseline yearly mortality**	12.3% [16]		
**CKD baseline yearly progression** 14.3% (13.6-15.0) [19]			
**RR of mortality by SP level**			For SA only
<2.5 mg/dL	0.95 (0.69-1.32) [17]	1.00 (0.96-1.24) [18]	1.00 (0.87-1.15)
2.5 mg/dL-3.0 mg/dL	1.00 (1.00-1.00) [17]	1.00 (0.96-1.24) [18]	1.00 (0.87-1.15)
3.0 mg/dL-3.5 mg/dL	1.15 (0.95-1.39) [17]	1.00 (0.93-1.07) [18]	1.00 (0.87-1.15)
3.5 mg/dL-4.0 mg/dL	1.32 (1.09-1.61) [17]	1.00 (0.93-1.07) [18]	1.00 (0.87-1.15)
4.0 mg/dL-4.5 mg/dL	1.34 (1.05-1.71) [17]	1.00 (1.00-1.00) [18]	1.00 (0.87-1.15)
4.5 mg/dL-5.0 mg/dL	1.83 (1.33-2.51) [17]	1.00 (1.00-1.00) [18]	1.00 (1.00-1.00)
5.0 mg/dL-5.5 mg/dL	1.90 (1.30-2.79) [17]	1.07 (1.01-1.14) [18]	1.00 (1.00-1.00)
5.5 mg/dL-6.0 mg/dL	1.90 (1.10-1.29) [17]	1.07 (1.01-1.14) [18]	1.02 (0.89-1.17)^‡^
6.0 mg/dL-7.0 mg/dL	1.90 (1.10-1.29) [17]	1.25 (1.17-1.34) [18]	
7.0 mg/dL-8.0 mg/dL	1.90 (1.10-1.29) [17]	1.43 (1.31-1.54) [18]	1.18 (1.02-1.36)^¥^
8.0 mg/dL-9.0 mg/dL	1.90 (1.10-1.29) [17]	1.67 (1.51-1.86) [18]	1.39 (1.21-1.60)
>9.0 mg/dL	1.90 (1.10-1.29) [17]	2.02 (1.76-2.27) [18]	1.39 (1.21-1.60)
**Utilities**			
Disease stage utility	0.71 [24]	0.61 [24]	

CA: calcium acetate; CC: calcium carbonate; CKD: Chronic Kidney Disease; CI: confidence interval; LC: lanthanum carbonate; RR: relative risk; SA: Sensitivity analysis; SP: Serum phosphorus.

^‡^from 5.5 mg/dL – 6.5 mg/dL.

^¥^from 6.6 mg/dL – 7.8 mg/dL.

**Figure 1:**
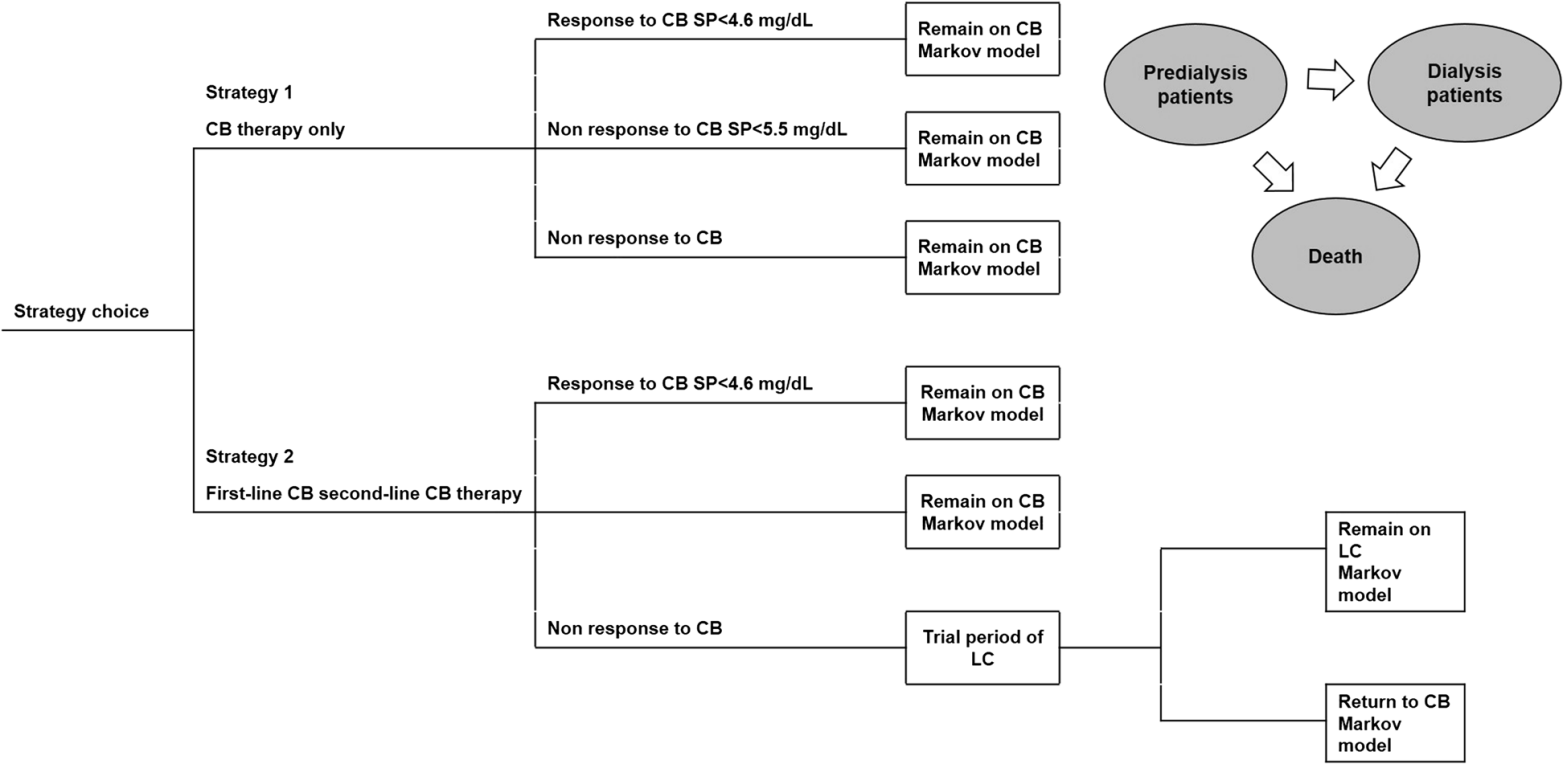
Decision analytical structure and Markov mode. CB: calcium-based binder; LC: lanthanum carbonate.

A initial decision tree with clinical pathways was used to identify subgroups of populations for further Markov model simulation (Figure 1). The three health states considered in the Markov model were: predialysis, dialysis and death. Patients were not allowed to transition from the ‘dialysis’ to ‘predialysis state’ (Figure 1). The duration of Markov cycles was established in one year, each patient remained at least one year in each condition. A half-cycle correction for outcomes and costs was applied to correct the fact that patients may progress to a different health state at any point during the one-year cycle [15].

### Mortality and progression rates

High SP levels are associated with mortality risk in both stage of disease (predialysis or dialysis), independent of renal function. Mortality rate for predialysis patients was estimated on 12.3% [16] adjusted with the relative risk related to SP levels [17]. Mortality rate for dialysis population was estimated from ERA-EDTA Annual Report and adjusted with SP levels [18]. Calcium binders efficacy is indirectly related to mortality rate as they are expected to control SP and reduce the associated of mortality [18]. CKD baseline progression rate included in this model was 14.3% per year [19]. Relative Risk of CKD progression was estimated to be 1.19 (1.10-1.29) per 1 g/dL of SP levels increase [19].

### Cost-effectiveness analysis

The model predicted the clinical benefits in dialysis free years and quality adjusted life years (QALY), and the associated costs with each intervention, providing the incremental cost-effectiveness ratio (ICER) of LC versus CB.

### Populations and strategies assessed

Two CKD populations (predialysis and dialysis) were considered to assess the cost-effectiveness of second-line LC treatment in patients previously treated with CB (calcium carbonate or calcium acetate) compared with the continued use of CB regardless of treatment response.

Strategy 1 was CB continued therapy regardless the treatment response for the whole cohort. Strategy 2 consisted on first-line treatment with CB, followed by a LC second-line for those patients identified as non-responders to CB in the decision tree.

Choice of second-line LC therapy initiation was modelled according to K/DOQI guidelines and panel expert opinion. Non-dialyzed patients with SP levels exceeding 5.5 mg/dL were treated in second line with LC [20]. Dialyzed patients non-responders to CB, started treatment with LC when SP exceeded 4,6 mg/dL [21]. Patients not achieving target SP with LC treatment after 8 weeks were switched back to CB. The model considered a SP target level of ≤4.6 mg/dL for predialyzed patients [3] and for dialyzed patients, based on the recommendations from an expert panel consulted, which was constituted by three Spanish nephrologists specialized in dialysis management.

### Clinical efficacy

Efficacy data of LC and CB were obtained from randomized clinical trials (Table 1). For predialysis patients, efficacy was based on pooled patient level data of predialysis and dialyzed patients due to low number of predialysis patients treated with LC. LC efficacy data in predialysis patients was taken from Sprague’s et al., a placebo-controlled study in dialysis, resulting in a SP reduction from baseline levels with LC (0.55 mg/dL vs 0.18 mg/dL with placebo, p = 0.02 for differences between groups) [20]. Only dialyzed patients (stage 3 and 4) with SP predialysis baseline values were included in order to increase homogeneity (n = 56 treated with LC). CB efficacy in predialysis was taken from a randomized controlled trial conducted in Spain, which compared Calcium carbonate with Calcium acetate efficacy in 28 predialysis patients during a time period of 24 months [22]. Patients baseline characteristics from both studies were comparable: SP levels 5.7 ± 1.3 mg/dL [22] vs 5.5 ± 1.0 mg/dL [20] (p = 0.37), age 59.0 ± 15.3 years [22] vs 61.8 ± 12.9 years [20] (p = 0.38) and glomerular filtration rate 20.5 ± 12.5 ml/min [22] versus 22.7 ± 6.7 ml/min [20] (p = 0.30). Efficacy data for CB and LC in dialyzed patients were based on a Phase III, randomized, active comparator-controlled trial evaluating the efficacy and safety of LC (n = 257 patients) versus calcium carbonate (n = 123) in CKD patients over a time period of 6 months [23]. The mean age of this population was 57 years (LC) and 58.4 years (calcium carbonate). On average, patients had received hemodialysis for 42.9 months (LC) and 43.8 months (calcium carbonate). The percentage of patients with residual renal function was 65.2% (LC) and 63.6% (calcium carbonate). Only patients with SP levels >5.58 mg/dL were randomized.

Long term efficacy was assumed as similar for predialysis and dialysis patients treated with LC and CB. Following current guidelines recommendations, target SP level for predialysis [3], in the model were <4.6 mg/dL. The clinical expert panel advised to consider the same target SP for the dialyzed patients.

### Utilities and adverse events

Quality of life (QoL) estimates were based on a published systematic review [24]. The results were averaged to result in utility estimate of 0.71 and 0.61 for predialysis and dialyzed patients. Data from clinical trials used to obtain efficacy data demonstrated that LC was associated to less adverse events rates than the comparator (47.4% vs 61.0% with placebo [20], or 77.7% vs 79.8% with calcium acetate [23]). However vomiting seemed to be associated with significantly increased rate in LC arm in predialysis patients (4.0%) [20], and dialysis patients (7.2%) [23], so it was decided to test influence of the potential affectation in patient QoL. Based on a published study, the model considered a utility decrement of 0.04082 for each vomiting episode [25].

### Perspective, time horizon, and discount rate

The analysis was performed for a Spanish public health care system perspective. Lifetime horizon (40 years maximum) was adopted for base case following all patients until death, applying a 3% annual discount rate for both, costs and health benefits, according to the last published recommendations [26].

### Costs

Based on the perspective, only direct health cost were included. All costs were expressed in euros (€), 2013 year value (Table 2).

**Table 2:** **Unitary cost (€, 2013)**

**Pharmaceutical costs**		**Presentation cost (ex-factory price including rebate)**	**Cost per gram (€/g)**	**Annual treatment cost**	
				***Predialysis***	***Dialysis***
Lanthanum carbonate [27]					
*Fosrenol® 750 mg*	90 chewable tablets	€167.86	€2.48	€1,702	€2,042
Calcium binders (average CC, CA) [27]				€49	€93
Calcium carbonate (CC)					
*Mastical® 1,250 mg*	60 chewable tablets	€2.09	€0.027	€30	€50
	90 chewable tablets	€2.97			
Calcium acetate (CA)					
*Royen® 1,250 mg*	60 chewable tablets	€7.13	€0.124	€68	€136
	120 chewable tablets	€3.91			
**Dialysis costs **[28]					€42,556

CA: Calcium Acetate; CC: Calcium Carbonate.

Daily Drug Doses (DDD) were based on expert panel recommendations. Drug costs were obtained from the Spanish General Council of Official Pharmaceutical Colleges catalogue [27]. Ex-factory prices adjusted with 7.5% mandatory deduction [28,29] were used. Annual costs for a dialyzed patient were estimated on €42,555.6 based on Ministry of Health estimations from a national health database [30]. No additional cost was considered for managing adverse effects (vomits). Prolonged dialysis care is related to the extended life of treated patients rather than to phosphate binder choice [31,32]. Dialysis costs in the added life years were classified as unrelated future costs and in line with previous cost-effectiveness analysis [14] were not included in the base case, but explored in sensitivity analysis.

### Sensitivity analysis

One-way and probabilistic sensitivity analysis were performed to test the robustness of the model and to determine the impact of uncertainty on the incremental cost-effectiveness ratio. The following parameters were varied: inclusion of unrelated future costs (long term dialysis costs), comparator (calcium carbonate only or calcium acetate only), time horizon (5 and 10 years horizon) and discount rate (0% and 6%)

Probabilistic analysis by a Montecarlo simulation was performed varying simultaneously the values for input parameters with a specific probability distribution for each of the parameters of interest. This process was repeated 1,000 times to provide a distribution of the model results. Costs and dose equivalence ratios were assumed to be lognormal distributed, binomial distributions were adopted for treatment response probabilities, normal distributions for relative risks and mortality rate of dialysis patients and beta distributions for health related utilities.

## Results

Over a lifetime horizon, LC achieved 4.653 QALYs per treated patient. With CB 4.579 QALYs were gained. Drug costs for LC therapy were €1,169, whereas for CB they were €468. The additional SP lowering effects of LC delayed CKD progression in LC responders, resulting in 108 dialysis free years gained. These dialysis free years resulted in large health care cost benefits because of the decrease of the dialysis costs with CB estimated in €4,576. Difference on lifetime total costs were € -3,875 for LC versus CB.

Second-line LC was associated with higher health benefits and also with costs savings, and therefore was identified as a dominant strategy over continuous CB treatment. The estimated costs and clinical benefits of the use of LC as second line therapy after therapy failure with CB in predialysis and dialysis patients to prevent CKD progression and mortality are shown on Table 3.

**Table 3:** **Cost-effectiveness results (cohort of 1,000 patients)**

	**Continuous CB**	**Second line LC**	**Difference**
**Therapy response**			
Number of responders in predialysis	445	658	213
Number of responders in dialysis	−60	−57	3
Total number of responders	385	601	216
**Health outcomes**			
Life Years	6,868	6,981	113
Dialysis free years	0	108	108
QALY’s	4,579	4,653	74
**Costs –(€, 2013)**			
Total costs (€ thousand)	€5,044	€1,169	€ -3,875
Drug costs (€ thousand)	€468	€1,169	€ 701
Dialysis costs (€ thousand)	€4,576	€0	
**Cost-effectiveness incremental ratio (ICER)**			
Cost per life-year gained (€)	Dominant		
Cost per QALY gained (€)	Dominant		
Net monetary benefit (€ thousand)	€6,092		

CA: Calcium Acetate; CB: Calcium Binder; CC: Calcium Carbonate; ICER: Incremental cost-effectiveness ratio; LC: Lanthanum Carbonate; QALY: Quality Adjusted Life Year.

Detailed results in terms of total costs and health benefits obtained for 1,000 patients followed lifetime, with each of the comparators are shown. Differences between therapeutic alternatives were calculated to estimate ICER. LC resulted a dominant strategy (more efficacious and less costly) compared to CB.

One-way sensitivity analysis results confirmed model and parameters robustness as LC continued being a dominant strategy in all the analysis performed (Table 4), apart from the inclusion of unrelated future dialysis costs, which provided an ICER of €45,554 per QALY gained. Although no specific threshold is officially established in Spain, this value is around one of the commonly accepted threshold of €45,000/QALY gained proposed by other authors for Spain [33].

**Table 4:** **One-way sensitivity analysis**

		**Continuous CB**	**Second line LC**	**Difference**	**ICER (€/QALY gained)**
**BASE CASE**					
	**QALYs**	**4,579**	**4,653**	**73.88**	**Dominant**
	**Costs (thousands)**	**€5,044**	**1,169**	**- €3,875**	
SA1	Time horizon (5 years)				
	QALYs	2,555	2,580	25.50	Dominant
	Costs (€ thousand)	€2,452	€835	- €1,616	
SA2	Time horizon (10 years)				
	QALYs	3,711	3,760	48.67	Dominant
	Costs (€ thousand)	€3,920	€1,028	- €2,892	
SA3	Included unrelated future dialysis costs				
	QALYs	4,579	4,653	73.88	€45,557
	Costs (€ thousand)	€127,149	€130,515	€3,336	
SA4	Dialysis target level 5 mg/dL				
	QALYs	4,579	4,658	79.30	Dominant
	Costs (€ thousand)	€5,044	€1,383	- €3,661	
S5	Annual Discount Rate (6%)				
	QALYs	3,846	3,903	56.95	Dominant
	Costs (€ thousand)	€4,122	€1,036	- €3,086	
S6	Annual Discount Rate (0%)				
	QALYs	5,598	5,498	99.48	Dominant
	Costs (€ thousand)	€6,346	€1,348	- €4,999	
S7	Only considering Acetate carbonate				
	QALYs	4,643	4,713	69.54	Dominant
	Costs (€ thousand)	€4,906	€1,324	- €3,582	
S8	Only considering Calcium carbonate				
	QALYs	4,511	4,590	79.02	Dominant
	Costs (€ thousand)	€5,223	€1,025	- €4,198	
S9	Dialysis mortality from Block 1998				
	QALYs	4,571	4,620	49.51	Dominant
	Costs (€ thousand)	€4,840	€1,170	-€3,670	
S10	Without utility decrement for vomiting				
	QALYs	4,579	4,653	73.93	Dominant
	Costs (€ thousand)	€5,044	1,169	- €3,875	

CA: calcium acetate; CB: Calcium Binder; CC: calcium carbonate; ICER: Incremental cost-effectiveness ratio; LC: lanthanum carbonate; QALY: Quality Adjusted Life Year.

**Figure 2:**
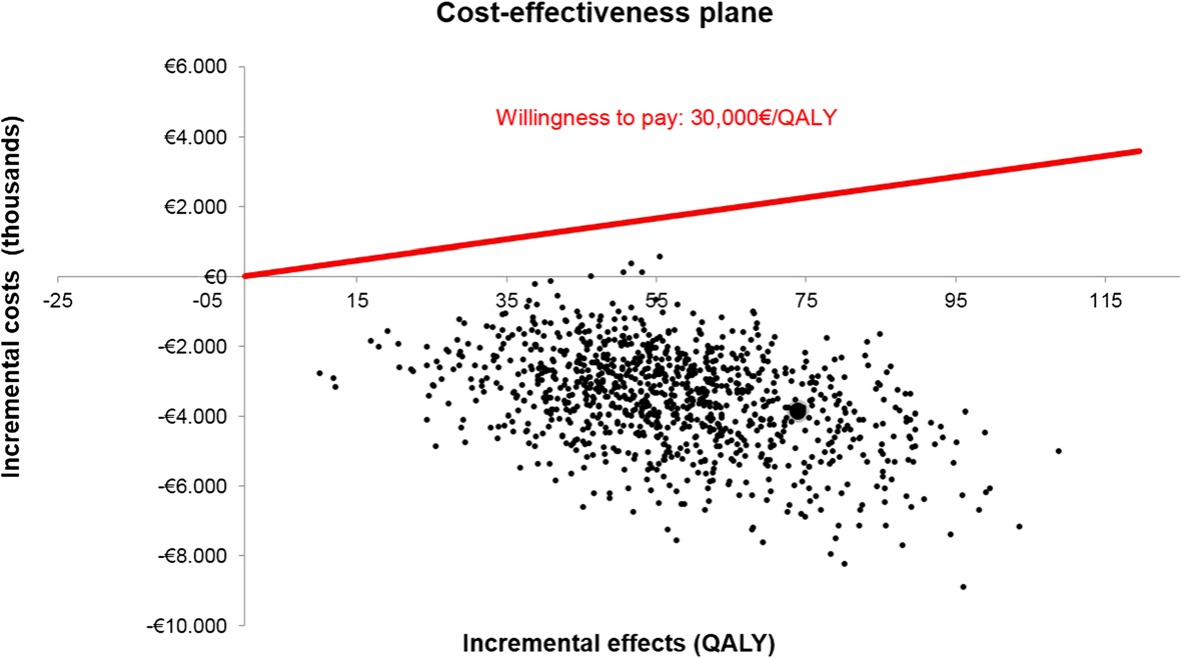
Cost-effectiveness plane. QALY: Quality Adjusted Life Year. The cost-effectiveness plane is the most common representation of the results of cost-effectiveness analysis results. The origin represents the standard alternative (calcium binders). Each point represents the ICER of each one of the 1,000 simulations conducted comparing the new alternative (LC) versus the standard one (CB). Most of the ICERs of LC versus CB resulted in lower costs and higher effectiveness, so points fell in the second quadrant, LC being classified as dominant option.

Time horizon was a parameter with great influence on results. Time horizon shortening had a positive effect on ICER, being the analysis with the shortest time horizon (5 years) the one with the lowest ICER. Figure 2 shown results of 1,000 interactions, performed on probabilistic sensitivity analysis, plotted on a cost-effectiveness plane. LC resulted a dominant option in 99.6% of the simulations. Assuming either a €45,000/QALY gained threshold [33] or an alternative €30,000/life year gained threshold [34], LC compared to CB would be a cost-effective strategy as second line treatment in 100% of simulations.

## Discussion

The dose of CB in CKD disease patients with hyperphosphatemia has been related with the severity of arterial calcification and death in these patients [35]. High calcium levels also stimulate the induction of a hypercoagulability site directly related to an increase in cardiovascular mortality risk [36-38] .

Although its relation with hypercalcemia, CB prescription in Spain has increased over recent years due to its lower price and the current healthcare budget restrictions in Spain [39].

Recent studies have suggested that LC treatment was associated with the reduced progression of aortic calcification in dialyzed patients [9]. However, additional clinical studies involving larger sample sizes and long-term follow-up are required to confirm this fact, and to rule out the possibility that the continuous LC administration might be generate any adverse events.

However, studies have demonstrated that prevention and delaying of end stage kidney disease lead to not only clinical benefits but also important cost savings because dialysis cost are high [40,41]. In addition, the cost-effectiveness of dialysis process has already been contested [42].

Despite of the great differences on pharmaceutical costs between LC and CB, in the present economic evaluation, second-line treatment with LC resulted a dominant option over CB therapy. The inclusion of unrelated future dialysis costs was associated with higher drug costs over a patient’s lifetime horizon than CB treatment, but the estimated ICER (€45,557/QALY) was just around one of the commonly accepted Spanish threshold (willingness to pay) [33]. Caution is necessary in comparing results with other economic evaluations due to differences in methods, setting and input parameters, as well as to differences in the alternatives used in the evaluations. However, the original model structure performed was used to perform an assessment of LC cost-effectiveness from a UK healthcare perspective [14] and also concluded that LC was a cost-effective strategy compared to CB therapy for the treatment of hyperphosphatemia, after failure of a CB first-line treatment.

Some limitations should be taken into account when interpreting findings derived from the present analysis. The most important were related to the limited efficacy data in predialysis patients and the potential uncertainty associated. However, this has already been widely discussed in the previous publication using the same model for UK setting [14].

Due to the lack of studies, epidemiological data related to mortality and CKD progression have been considered from studies conducted in other countries other than Spain. Nevertheless, based on their experience and knowledge the expert panel considered that these data were representative for the Spanish population.

QoL is highly related to social preferences. The utilities values used in the present model derived from a systematic review in CKD population [24] which assessed studies on different settings and countries. No study specifically referring to a Spanish population with CKD was found by authors, so international values were applied, based on the assumption that the sources used were representative of the Spanish population as they have been taken from European publications. Similarly, the utility decrement due to vomiting as an adverse event related to LC was based on a study on non-small lung cancer [25], because specific data in populations with renal disease were unavailable. Furthermore, the influence of these parameters on the cost-effectiveness outcomes was small. Influence of adverse events other than vomiting was not tested. Available evidence about safety profile of LC [9,23] suggested an equivalent or higher tolerability than CB, so the results shown could be considered underestimating the total cost of CB.

Although they have been widely demonstrated [5], the potential deleterious effects of CB were not modelled. The expert panel considered that against recommendations for hyperphosphatemia treatment of current clinical guidelines in Spain, CB are not always prescribed in clinical practice as first treatment option, due to these negative effects.

Pill burden reduction associated to LC therapy compared to CB could improve adherence and therapy compliance [14]. CKD lack of adherence to treatment in Spain has been highlighted by an important observational study of 121 hemodialysis patients [43]. The lack of adherence to treatment not only prevents the achievement of control targets but may additionally represent a financial burden to the health system and pose a major obstacle to effective treatment [44] In this sense, LC oral powder has already demonstrated its efficacy, and its positive effect on adherence to treatment in CKD patients [45].

However, due to the lack of information along with the difficulties to quantify adherence, influence of pill burden on QoL or drug efficacy was not included in the model representing a conservative approach for LC, because increase on patients QoL could be potentially derived from its inclusion. Further investigations on the LC pill burden reduction effect will be interesting to be considered in future economic evaluations as better adherence would be expected to impact positively on improved efficacy.

The inclusion or exclusion of unrelated future costs is the topic of a long-standing and as-of-yet unresolved discussion. Unrelated future costs were excluded from the base-case of the present analysis but tested on sensitivity analysis. This demonstrated a sizeable influence on the cost-effectiveness results although the inclusion of these costs is an area of important debate in economic evaluations [46].

Although the present results could not be considered as definitive due to the limitations mentioned, they could provide useful information for clinicians and decision makers. Overall, we attempted to use conservative assumptions and approaches for each of the limitations mentioned, therefore findings provided by the present model can be considered as conservative. It would be interesting to confirm preliminary results in further evaluations, but many times it is not feasible, so this analyses aimed to increase the scientific knowledge and any effort on this way must be welcomed, meanwhile the lack of reliable local data avoids a model update.

Results from cost-effectiveness studies along with the higher mortality rate and higher costs (related to dialysis) associated to CB, have also to be taken into account not only by clinicians but also by decision makers in order to protect the National Health Service.

## Conclusions

The findings of the present model suggested that in Spain LC therapy for second line treatment of hyperphosphatemia in CKD patients was a dominant strategy compared to continuous CB treatment. This important finding should be taken into account when choosing a phosphate binder treatment for hyperphosphatemia associated to CKD.
